# Peer Review of “Chaotic and Stochastic Components in an Influenza Surveillance Series: Nonlinear Dynamics and Predictive Modeling Study”

**DOI:** 10.2196/101468

**Published:** 2026-06-05

**Authors:** Yulia Bobrova

**Affiliations:** 1 Saint Petersburg State Electrotechnical University, St. Petersburg, Russian Federation

**Keywords:** epidemiology, epidemiologic methods, epidemiological monitoring, chaos theory, topological data analysis, autoregressive conditional heteroskedasticity

*This is a peer-review report for “Chaotic and Stochastic Components in an Influenza Surveillance Series: Nonlinear Dynamics and Predictive Modeling Study.”*

## Round 1 Review

The manuscript [1] under review presents an empirical methodology for studying stochastic chaos in epidemiological data by combining topological data analysis, topological machine learning, and nonlinear time series analysis to decompose influenza dynamics into deterministic chaotic and stochastic components down to the noise of independent and identically distributed random variables.

I recommend that the authors address the following shortcomings to improve the manuscript before publication.

1. The estimated largest Lyapunov exponent is approximately 0.001, which the authors characterize as “weak chaos.” However, such low values may be statistically indistinguishable from colored noise or stochastic processes with long-range dependencies. The authors should conduct additional tests using surrogate data to reliably confirm the deterministic chaotic nature of the reconstructed attractor.
2. The authors should elaborate on how identifying stochastic chaos improves decision-relevant forecasting compared to established time series models in disease outbreak scenarios, including a discussion of lead times, calibration, and uncertainty quantification.
3. The study reports high explanatory power for the full model but does not compare its performance to benchmark forecasting methods. Including a comparative analysis with traditional epidemiological or statistical models would better contextualize the added value of the topological machine learning approach and strengthen claims of methodological superiority.

### Minor Comments

Ensure consistent hyphen usage in compound terms (“time-series” vs “time series”).

## References

[1] dos Santos Goncalves CP, Rouco C (2026). Chaotic and Stochastic Components in an Influenza Surveillance Series: Nonlinear Dynamics and Predictive Modeling Study. JMIRx Med.

